# Mice lacking GPR3 receptors display late-onset obese phenotype due to impaired thermogenic function in brown adipose tissue

**DOI:** 10.1038/srep14953

**Published:** 2015-10-12

**Authors:** Grzegorz Godlewski, Tony Jourdan, Gergő Szanda, Joseph Tam, Resat Cinar, Judith Harvey-White, Jie Liu, Bani Mukhopadhyay, Pál Pacher, Fong Ming Mo, Douglas Osei-Hyiaman, George Kunos

**Affiliations:** 1 Laboratory of Physiologic Studies , National Institute on Alcohol Abuse and Alcoholism of the National Institutes of Health, 5625 Fishers Lane, RM 2S-18, MSC-9413, Bethesda, Maryland 20892-9413, U.S.A.; 2 Laboratory of Cardiovascular Physiology and Tissue Injury, National Institute on Alcohol Abuse and Alcoholism of the National Institutes of Health, 5625 Fishers Lane, RM 2N-17, MSC-9413, Bethesda, Maryland 20892-9413, U.S.A

## Abstract

We report an unexpected link between aging, thermogenesis and weight gain via the orphan G protein-coupled receptor GPR3. Mice lacking GPR3 and maintained on normal chow had similar body weights during their first 5 months of life, but gained considerably more weight thereafter and displayed reduced total energy expenditure and lower core body temperature. By the age of 5 months GPR3 KO mice already had lower thermogenic gene expression and uncoupling protein 1 protein level and showed impaired glucose uptake into interscapular brown adipose tissue (iBAT) relative to WT littermates. These molecular deviations in iBAT of GPR3 KO mice preceded measurable differences in body weight and core body temperature at ambient conditions, but were coupled to a failure to maintain thermal homeostasis during acute cold challenge. At the same time, the same cold challenge caused a 17-fold increase in Gpr3 expression in iBAT of WT mice. Thus, GPR3 appears to have a key role in the thermogenic response of iBAT and may represent a new therapeutic target in age-related obesity.

Obesity poses a major global healthcare burden that is no longer confined to developed nations. A recent report estimates that almost a third of the young and adults worldwide are overweight or obese^1^ and this fraction is projected to further rise as a result of an aging society^2^,^3^,^4^,^5^,^6^. By 2050, the number of U.S. adults aged 65 and over is expected to more than double from 40.2 million to 88.5 million^7^.

Despite the overall remarkable progress in understanding bioenergetic pathways, the question as to why aging predisposes to weight gain remains inadequately addressed. Adult or late-onset obesity has been observed in mice lacking platelet activating factor^8^, type-3 adenylyl cyclase^9^, insulin receptor in brown adipose tissue^10^, in human renin gene transgenic mice^11^ or has been experimentally induced in ovariectomized young female rats^12^. Age-related weight gain has been consistently associated with impaired thermogenesis due to a programmed loss of function of interscapular brown adipose tissue (iBAT)^8^,^13^,^14^, which shifts surplus energy to visceral and subcutaneous fat depots for storage^12^,^14^,^15^. Yet, age-dependent factors controlling iBAT function remain largely unknown.

GPR3 is a class A orphan member of the G protein-coupled receptor (GPCR) superfamily, homologue of the cluster that includes GPR6 and GPR12, whose native ligands are still unknown^16^,^17^,^18^,^19^,^20^. A synthetic small molecule was recently identified as a specific surrogate agonist of human GPR3^21^,^22^. GPR3 is highly expressed in oocytes where it has been linked to maintaining oocyte meiotic arrest^23^,^24^,^25^,^26^,^27^,^28^,^29^,^30^ and ovarian failure^31^,^32^,^33^. GPR3 is also highly expressed in brain areas implicated in neurodegenerative diseases^34^,^35^. The possible role of GPR3 in metabolic and aging effects, particularly in relation to weight gain, has not been explored. Our interest in GPR3 was initially driven by its sequence homology with two existing cannabinoid receptors^36^, which made it a potential candidate in our search for an as yet unidentified third cannabinoid receptor. We were surprised, however, to find a novel and unexpected phenotype, which has provided a new direction to our study. Here we report an unexpected link between aging and obesity *via* GPR3. Mice lacking GPR3 display late-onset obesity associated with a reduction in uncoupling protein 1 protein level in iBAT and thermogenesis.

## Results

### Older Gpr3 KO mice have obese phenotype

**Figure 1:**
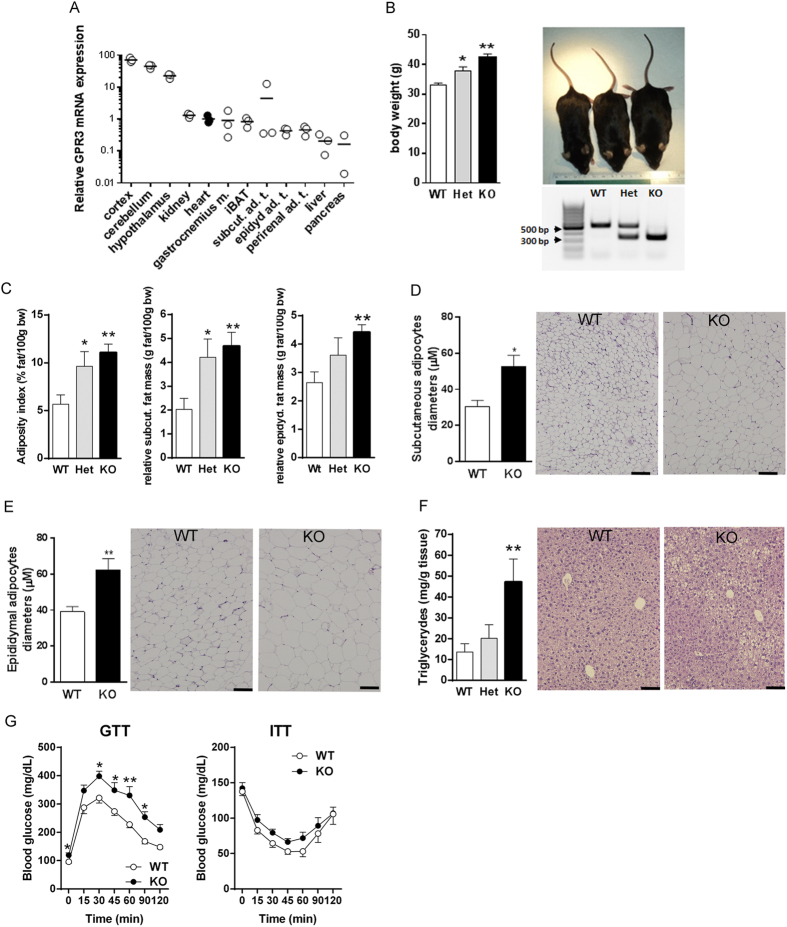
Phenotype differences among GPR3 mice. (**A**) Tissue expression profile of GPR3 mRNA in 12- week-old wild type male mice. Each circle represents a single value relative to heart (filled circles). (**B**) Body weights of 1-year-old male wild type (WT), heterozygous (Het) and knockout (KO) mice along with genotyping results and representative images of WT (33.29 g), Het (36.52 g) and KO (41.48 g) mice. Values are mean ± s.e.m of n = 16–19 animals. *P < 0.05, **P < 0.01, ***P < 0.001 compared to age-matched WT littermates. (**C**) Adiposity index, relative mass of subcutaneous fat and epididymal fat, (**D**) subcutaneous adipocyte diameters, (**E**) epididymal adipocyte diameters and (**F**) liver triglyceride level in GPR3 WT, Het and KO mice. Values are mean ± s.e.m of n = 7–14 samples. *P < 0.05, **P < 0.01 compared to age-matched WT littermates. Panels D-F are accompanied by the representative H&E stained tissue sections. Pictures were taken at 10x magnification (scale bar indicates 100 μm). (**G**) Glucose tolerance (GTT) was tested after intraperitoneal (i.p.) injection of 1.5 g/kg glucose (at 0 min; (**A**) to the overnight-fasted 12-month-old male GPR3 WT and KO mice. On the following day, mice were fasted for 6 hour and received 0.75 mU/g insulin i.p. at time 0 (insulin sensitivity test; ITT). Changes in blood glucose level were monitored for 2 hours. Values are mean ± s.e.m of n = 8–14 samples. *P < 0.05, **P < 0.01 compared to age-matched WT littermates.

*Gpr3* is expressed in several metabolically active peripheral tissues, although at lower levels than in the central nervous system (CNS) (Fig. 1A), and its partial or total deletion leads to genotype-specific changes in body weights of older (12-month-old) mice (Fig. 1B). Thus, over the course of one year, GPR3 heterozygous (Het) and knockout (KO) mice maintained on a regular mouse chow gained on average ~15% and ~30% more weight, respectively, than their age-matched wild type (WT) littermates and were visually distinguishable from each other (Fig. 1B). Differences in body weights among 12-month-old GPR3 WT, Het and KO females were also significant, but less prominent than in males (27.31 ± 1.14 g, 27.39 ± 0.83 g and 30.93 ± 1.04 g, respectively; P < 0.05, n = 12–25). Therefore, males were used in further analyses.

The adiposity indices in older GPR3 Het and KO male mice rose 1.7 ± 0.3-fold and 2.0 ± 0.1-fold, respectively, when compared to WT littermates (Fig. 1C). The most prominent differences were observed in subcutaneous (Fig. 1D) and epididymal (Fig. 1E) fat depots, which accounted for the majority of the adiposity and weight gain. In agreement with appearances on histological sections, subcutaneous and epididymal adipocytes were noticeably larger in 12-month old GPR3 KO mice than in age-matched WT littermates (Fig. 1C). Hematoxylin and eosin (H&E) staining of liver sections from older GPR3 KO mice revealed fat droplet accumulation, which was paralleled by a marked increase in hepatic triglyceride content (Fig. 1C). Surprisingly, deletion of GPR3 had no effect on serum metabolic markers in non-fasting 12-month-old mice. One notable exception was leptin, whose high concentration in GPR3 KO mice directly correlated with their increased adiposity (Table 1). Also, after overnight fasting, basal blood glucose concentration in older GPR3 KO mice was significantly higher than in their WT littermates, and GPR3 KO showed a marked impairment of their glucose tolerance (GTT; Fig. 1G, left panel), as reflected by a higher AUC value than in the WT counterparts (353.63 ± 34.02 and 248.02 ± 11.64 hour x mg/dL, respectively, P < 0.05; n = 11–14). Additionally, GPR3 KO mice tended to have lower sensitivity to insulin, although this did not reach statistical significance (ITT; Fig. 1G, right panel).

### Older GPR3 KO mice have reduced total energy expenditure

**Figure 2:**
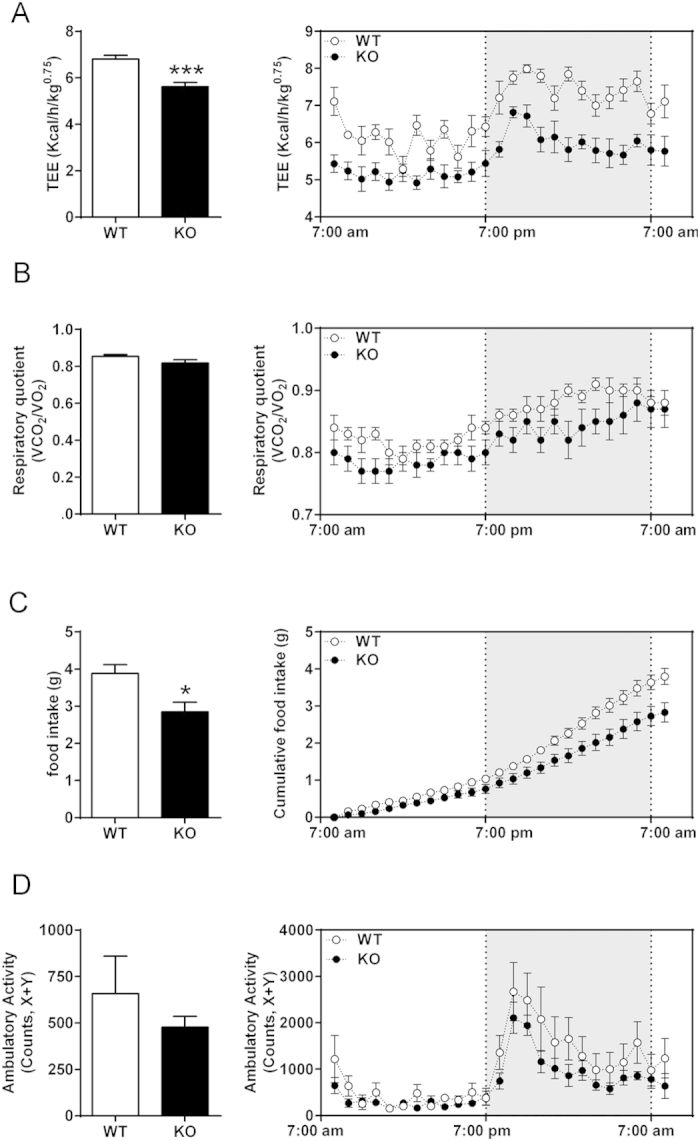
Metabolic profiles of older GPR3 mice as analyzed by means of indirect calorimetry. The O_2_ consumption and CO_2_ production were monitored by indirect calorimetry in older (12-month-old) male GPR3 wild type (WT) and knockout (KO) mice and presented as average of a 24-hour recording period (left panels) or as hourly observations (right panels; shaded area indicates lights off). Shown are total energy expenditure (TEE; (**A**)), respiratory quotient (RQ; (**B**)), food intake (**C**) and ambulatory activity (**D**). Values are mean ± s.e.m of n = 5–8 samples *P < 0.05, ***P < 0.001 compared to age-matched WT littermates.

Indirect calorimetry revealed a marked and sustained decrease in the total energy expenditure in older GPR3 KO mice (TEE; Fig. 2A) without a significant change in their respiratory quotient (RQ; Fig. 2B). The reduction in TEE was associated with concomitant drops in oxygen consumption and carbon dioxide production in the GPR3 KO group (Figure S1). Food intake was also lower in GPR3 KO mice than in their WT littermates (Fig. 2C), while, importantly, the ambulatory activity remained similar between the genotypes (Fig. 2D). These metabolic differences were similar during the light and dark phases (Fig. 2, right panels) and are in harmony with the obese phenotype.

### GPR3 KO mice display late onset obese phenotype

**Figure 3:**
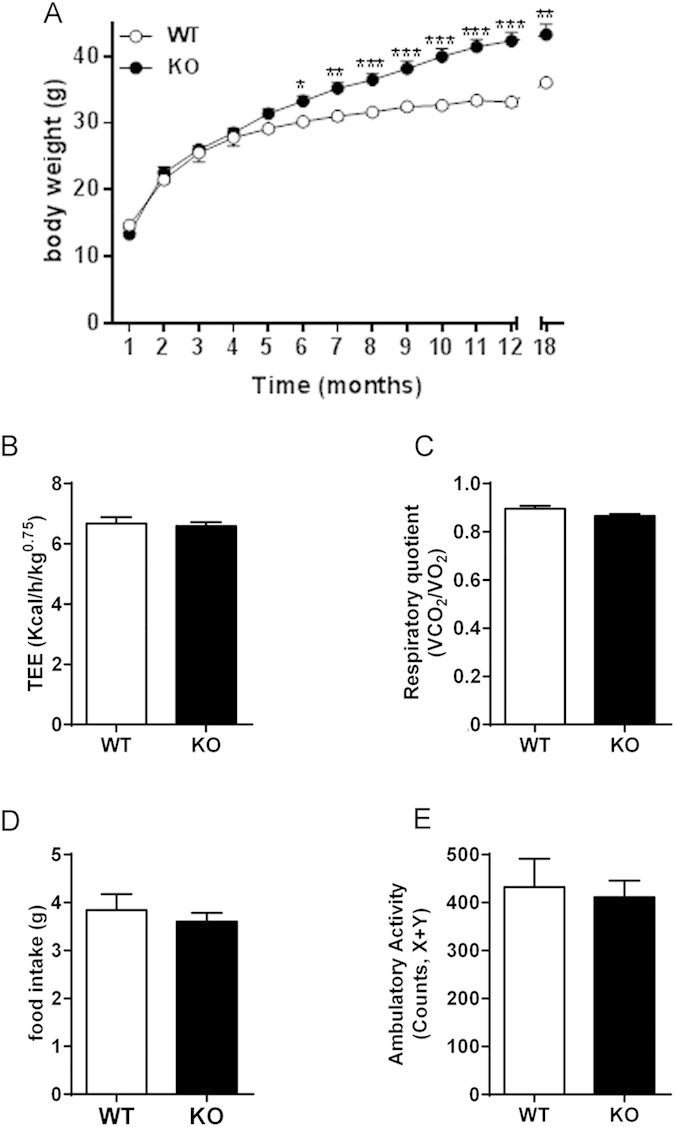
GPR3 KO mice display late onset obese phenotype with unchanged metabolic profile until adulthood. (**A**) Body weight monitoring for 18 months in male GPR3 wild type (WT) and knock out (KO) mice. Values are mean ± s.e.m of n = 7–23 animals. *P < 0.05, **P < 0.01, ***P < 0.001 compared to the age-matched WT littermates. (**B**–**E**) Results of the monitoring of O_2_ consumption and CO_2_ production by indirect calorimetry in adult (5-month-old) male GPR3 wild type (WT) and knockout (KO) mice. Data are presented as average of a 24-hour recording period. Shown are: total energy expenditure (TEE; (**B**)), respiratory quotient (RQ; (**C**)), food intake (**D**) and ambulatory activity (**E**). Values are mean ± s.e.m of n = 6–8 samples.

To examine the temporal dynamics of the GPR3 KO phenotype, we monitored the body weight of standard chow-fed mice for up to 18 months. As shown in Fig. 3A, body weights were not different between WT and GPR3 KO mice during the first 5 months of life, but GPR3 KO mice gained considerably more weight thereafter. At the age of 5 months, both groups had similar values of TEE, RQ, food intake and ambulatory activity (Fig. 3B–E). They also had similar fecal triglyceride content (Figure S2), suggesting comparable intestinal absorption capacities. As animals further advanced in age, GPR3 KO mice began to gain considerably more weight. In order to better understand why GPR3 KO mice develop obesity later in life we performed additional studies on 5-month-old (adult) animals, as this was the latest time point before the obese phenotype became apparent.

### Adult GPR3 KO mice have impaired glucose uptake into iBAT

**Figure 4:**
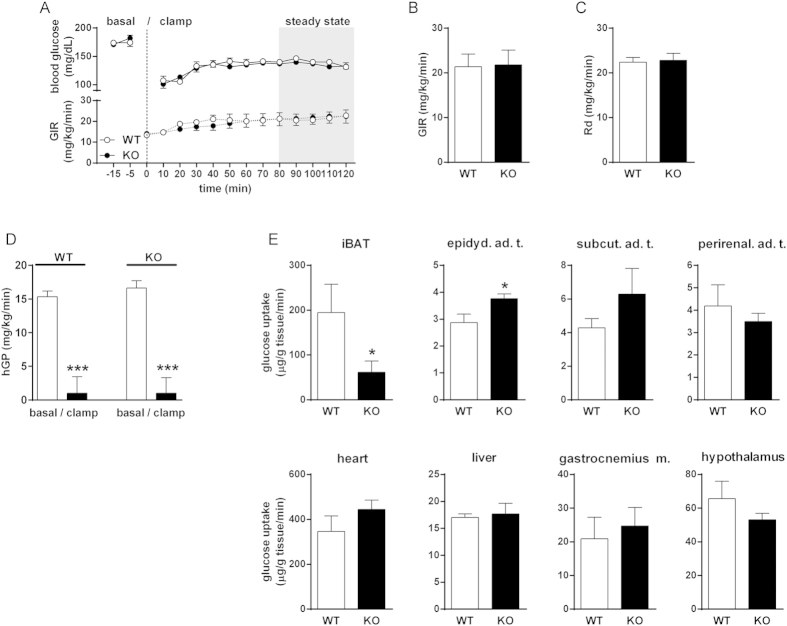
Glycemic control in adult GPR3 mice. (**A**–**E**) Hyperinsulinemic euglycemic clamps were performed in conscious, unrestrained 5-month-old male GPR3 WT and KO mice. Time course of blood glucose levels and glucose infusion rates (GIR; (**A**)) during the course of clamps; Glucose Infusion Rate (**B**), whole body glucose clearance (Rd; (**C)**), levels of hepatic glucose production (hGP; (**D**) were measured during the steady-state period of the clamp. (**E**) Glucose uptake into individual tissues: interscapular brown adipose tissue (iBAT), epididymal (epidyd. ad. t.), subcutaneous (subcut ad. t.) and perirenal adipose tissues (ad. t.), heart, liver, gastrocnemius muscle (gastrocnemius m.) and hypothalamus. Tissue uptake was determined as described in materials and methods. Values are mean ± s.e.m of n = 6–11 samples (except hypothalamus for which n = 5–7). ***P < 0.001 compared to age-matched WT littermates.

We first performed hyperinsulinemic euglycemic clamp experiments to examine tissue-specific differences in glucose uptake in 5-month-old mice. At this age, WT and GPR3 KO animals still had similar body weights (31.55 ± 1.02 g and 31.06 ± 1.15 g, respectively; n = 6–7). Pre-clamp basal blood glucose levels (Fig. 4A), glucose infusion rates (GIR) and blood glucose levels during the clamp (Fig. 4A–B), whole body glucose clearance (Rd; Fig. 4C) and insulin-induced suppression of hepatic glucose production (hGP; Fig. 4D) were also similar between adult GPR3 genotypes. Yet, there were some prominent differences between WT and GPR3 KO mice in the glucose uptake in individual tissues (Fig. 4E). Particularly noteworthy was a 2-fold lower glucose uptake in the iBAT of GPR3 KO mice compared to their WT counterparts. In contrast, glucose uptake was significantly higher in epididymal adipose tissue of GPR3 KO mice, while it remained similar between the groups in the subcutaneous and perirenal fat depots, liver, and gastrocnemius muscle and in the hypothalamus (Fig. 4E).

### Molecular changes in iBAT of adult GPR3 KO mice precede the decrease in core body temperature of older mice

**Figure 5:**
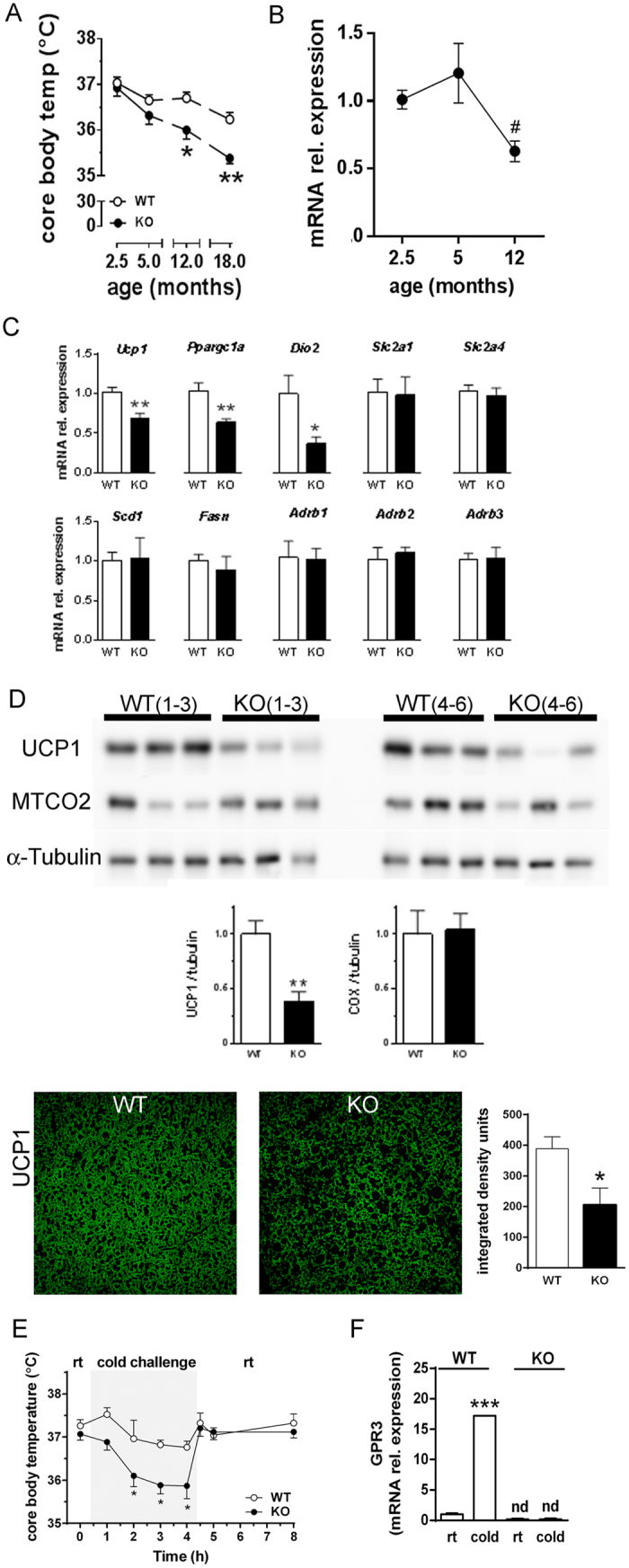
Thermogenic features of adult GPR3 mice. (**A**) Core body temperature was recorded in 2.5-, 5-, 12- and 18-month-old male GPR3 wild type (WT) and knock out (KO) mice. Values are mean ± s.e.m of n = 6–13 samples. *P < 0.05, **P < 0.01 compared to age-matched WT littermates. (**B**) Relative abundance of GPR3 transcript in iBAT obtained from adult male GPR3 WT at different ages. Values are mean ± s.e.m of n = 4–6 animals. ^#^P < 0.05, compared to 5-month-old mice. (**C**) Relative abundance of indicated transcripts in interscapular brown adipose tissue (iBAT) of adult (5-month-old) male GPR3 WT and KO mice at room temperature. Values are mean ± s.e.m of n = 3–9 samples. *P < 0.05, **P < 0.01, compared to the age-matched WT littermates. (**D**) The UCP1, MTCO2 and α-tubulin (tubulin) proteins detected in iBAT from two groups of male GPR3 WT and KO mice (1–3 and 4–6). Protein sizes corresponding to UCP1, COX and α-tubulin were 33 kDa, 25 kDa and 52 kDa, respectively. Representative fluorescent immunostaining images against UCP1 in iBAT of GPR3 WT and KO mice. Pictures were taken at 10× magnification (scale bar indicates 100 μm). Western blot band and staining intensities were calculated as described in Material and Methods section and presented as bar graphs next to images. Values are mean ± s.e.m of n = 6–8 samples. *P < 0.05, **P < 0.01 compared to age-matched WT littermates. (**E**) Changes in core body temperature in 5-month-old male GPR3 WT and KO mice undergoing cold challenge. Body temperature was measured at ambient conditions for baseline as well as 1, 2, 3 and 4 hours after the exposure to cold (4 °C, cold challenge) and 0.5, 1 and 4 hours during the recovery period at room temperature (rt). Values are mean ± s.e.m of n = 5–6 samples. *P < 0.05 compared to age-matched WT littermates. (**F**) Relative abundance of GPR3 transcript in iBAT obtained from adult male GPR3 WT and GPR3 KO mice obtained at room temperature (rt) and at the 4^th^-hour of cold challenge (cold). Values are mean ± s.e.m of n = 3–8 samples. ***P < 0.001, compared to age-matched GPR3 WT littermates at room temperature (rt). GPR3 transcripts were not detectable (nd) in GPR3 KO mice. All target genes in panels (**B**,**C**,**F**) were corrected via reference *Rpl*19 mRNA.

We did not observe substantial differences in core body temperature during the first 5 months of life, although body temperature tended to decline faster with time in GPR3 KO than in WT mice. By the time animals reached 12 months of age, body temperature was significantly lower in GPR3 KO than in WT mice and it remained lower 6 months later (Fig. 5A). This was accompanied by lower iBAT norepinephrine levels and lower serum thyroxine (T4), but not triiodothyronine (T3) and thyroid-stimulating hormone (TSH) concentrations in 12-month old GPR3 KO mice (Figure S3). Downregulation of Gpr3 gene expression was also noticeable in 12-month-old WT mice (Fig. 5B).

At 5 months of age, GPR3 KO and WT mice had comparable tissue norepinephrine levels and serum concentrations of T3, T4 and TSH (Figure S3). Yet, in spite of normal resting core body temperature, iBAT in adult GPR3 KO mice already displayed some molecular changes, which were in harmony with and predictive for the loss of thermogenic function at a later age. Mice lacking GPR3 had significantly lower basal expression of uncoupling protein 1 (UCP1), peroxisome proliferator-activated receptor-γ coactivator 1 alpha (PGC1α) and type 2 iodothyronine deiodinase (Dio2), with no change in genes encoding for adrenergic receptors, glucose transporters, fatty acid synthase or stearoyl-CoA desaturase (Fig. 5C). As evidenced by Western blot, iBAT of GPR3 KO mice also had a markedly reduced UCP1 content with no difference in the level of cytochrome c oxidase subunit II (MTCO2) protein. This observation was further supported by their lower UCP1 fluorescence-labeling intensity of iBAT sections (Fig. 5D).

In order to verify if decreased UCP1 protein in iBAT had a physiological impact on adult GPR3 KO mice, we temporarily placed them in a cold environment (4 ˚C) and examined the ability to defend their core body temperature after acute cold exposure. As indicated in Fig. 5E, WT mice were able to successfully maintain their physiological temperature in cold chambers for the duration of experiment. In contrast, despite having normal core body temperature at ambient condition, comparable iBAT norepinephrine levels and serum markers of thyroid function between the groups during the cold exposure (Figure S4), 5-month-old GPR3 KO mice exhibited an impaired tolerance to cold, which was manifested by a progressive decrease in their core body temperatures during the cold challenge. At the end of the cold session, body temperature in GPR3 KO mice was lower by an average of 0.9 °C than in the WT group, whereas body temperature of adult GPR3 KO mice immediately returned to pre-exposure levels once animals were transferred to their home cages at ambient conditions (Fig. 5D).

Acute cold exposure had a robust effect on *GPR3* mRNA expression in iBAT of WT mice, which increased 17 times relative to the level in ambient conditions (Fig. 5E). In contrast, the expression of *Gpr6* and *Gpr12* remained unchanged (Figure S5), whereas changes in other genes of interest during the cold challenge were rather modest and followed the same pattern in both groups. One notable exception was a higher, possibly compensatory increase in *Dio2* gene expression in GPR3 KO relative to that in WT mice (Figure S6).

The above results indicate that 5-month old GPR3 KO mice, while maintaining normal core body temperatures at ambient conditions, are compromised in their ability to defend thermal homeostasis during the cold challenge.

## Discussion

Aging has been inextricably associated with metabolic changes that predispose a person to obesity and related pathologies. Despite the overlapping signaling pathways, factors that link aging with obesity remain largely unknown.

In the present study, we have explored the effects of orphan GPR3 ablation on energy metabolism in mice. This receptor was previously implicated in premature ovarian aging^31^,^32^,^33^ and neurodegenerative disorders^34^,^35^, both of which are age-related pathologies. Here we report for the first time that male mice lacking GPR3 display late-onset obesity when maintained on regular chow diet. Body weights and metabolic profiles were not different among male GPR3 KO and WT groups during the first 5 months of life, thereafter body weights increased systematically and in a genotype-specific manner, so that by the age of 12 months, GPR3 KO mice were considerably heavier than their WT littermates, due to a substantially higher adiposity index and related to impaired thermogenesis. A gene dosing effect was also evident in the intermediate phenotype of heterozygous animals. Female GPR3 KO mice gained considerably less weight than males, indicating that premature ovarian aging is unlikely to be responsible for the obese phenotype and, if anything, may in fact limit the expression of this phenotype in female mice.

Our data are consistent with previous studies indicating that aging increases the susceptibility to adiposity and obesity^37^,^38^. Young rodents have a natural propensity to store both protein and fats when growing to maturity^39^. However, protein deposition eventually gives way to fat accumulation as animals age^40^. While 2.5 and 5-month old GPR3 KO mice weighed the same as their age-matched wild-type littermates, by the age of 12 months they had a pronounced obese phenotype, which was retained till the end of the study 6 months later. This indicates that GPR3 is a critical factor in protecting against fat deposition in the later stages of life. Enhanced fat accumulation in the subcutaneous and epididymal depots and in the liver of 12-month old GPR3 KO mice was metabolic in nature, as evidenced by the substantial decline in TEE, O_2_ consumption, CO_2_ production and core body temperature. Although 12-month old GPR3 KO mice also had lower food consumption, this parameter alone was not apparently sufficient to offset the decline in the body’s lower energy demand, resulting in a progressive increase in adiposity over time. Additionally, GPR3 KO mice did not differ from WT littermates with regard to their locomotor activity, therefore ruling out hypomotility as the primary cause of weight gain in the knockouts.

In spite of being overweight, the metabolic profile of 12-month old GPR3 KO mice did not display classic systemic features of the metabolic syndrome. Ablation of GPR3 did not cause systemic hyperglycemia, hyperinsulinemia, dyslipidemia, liver damage or alteration of major of metabolic markers under non-fasting conditions. As reflected by GTT and ITT tests, 12-month old GPR3 KO mice were glucose-intolerant, but remained insulin-sensitive, compatible with a state of lower energy utilization.

One might wonder which organ accounts for the decreased energy expenditure in the absence of GPR3. The first clue came from running hyperinsulinemic euglycemic clamp experiments, which revealed a major decline in glucose uptake by iBAT in 5-month old GPR3 KO mice. Surprisingly, only a handful of studies have addressed the possible involvement of iBAT dysfunction in age-related weight gain. These studies have shown that metabolic capacity of iBAT was diminished with age and related to a loss of multilocular cell organization^12^,^14^,^15^ and that the reduced browning of fat was associated with hypothyroid-like abnormalities, impaired mitochondrial function and lower response to cold or to β-adrenoceptor agonists^8^,^13^,^14^,^41^. As a consequence, animals develop glucose-intolerance but often remain insulin-sensitive^10^,^12^,^15^,^42^,^43^. Although iBAT norepinephrine levels and serum T4 concentrations were lower and likely contributed to metabolic changes in 12-month old GPR3 KO mice, one could not determine whether these changes were the cause or the consequence of an obese phenotype. Therefore, we focused on studying iBAT function in 5-month-old mice before the onset of an obese phenotype. In spite of similar physiology and metabolic characteristics, clear differences in iBAT function were already detectable at this age. In addition to decreased glucose uptake, iBAT of 5-month old GPR3 KO mice had reduced baseline expression of thermogenic genes accompanied by significantly lower level of UCP1 but not MTCO2 protein, compared to age-matched controls. This pattern suggests that adult GPR3 KO mice have an impaired ability to generate heat by non-shivering thermogenesis due to a lower level of UCP1, while still retaining the overall mitochondrial oxidative apparatus. Thermogenic activity of iBAT appeared sufficient to maintain thermal homeostasis under ambient conditions, yet it was inadequate to adapt to cold, which manifested in a marked and progressive reduction in body temperature during the cold exposure. The important role of the loss of GPR3 in this phenotype was further indicated by the finding that *Gpr3* expression in iBAT was robustly increased in ‘cold-resistant’ WT mice during the 4-hour cold exposure, much more than any other gene screened in this study, including the *Gpr3* homologues *Gpr6* and *Gpr12* whose expression did not change at all. This implies that *Gpr3* is rapidly activated in response to a cold stress and may serve as an essential and specific contributor to adaptive thermogenesis.

Thermogenesis is a vital component of the homeostatic repertoire, which maintains normal body temperature in the face of diverse environmental challenges^41^. It is regulated by an intricate neuronal network in the CNS, which receives afferent inputs from cutaneous and core body thermoreceptors and communicates with iBAT *via* sympathetic innervation^44^ and the hypothalamic-pituitary-thyroid axis^45^. Here we show for the first time that mice lacking GPR3 have impaired thermogenic function of the iBAT. An important question still remains whether GPR3 exerts its effects by central or peripheral mechanisms. Serum TSH concentration and tissue norepinephrine levels are relevant indicators of centrally driven thyroid signaling and sympathetic activity. As changes in the molecular reorganization of iBAT of 5-month-old GPR3 KO mice were not accompanied by a difference in either of the above, peripheral rather than central GPR3 may play a dominant role in triggering the iBAT dysfunction. These findings also suggest that local catecholamine and/or thyroid function might be impaired at the cellular level as a result of GPR3 ablation. One cannot entirely neglect the contribution of central GPR3 in the control of temperature and weight as *Gpr3* mRNA expression was over 10-times higher in brain than in peripheral tissues. Nevertheless, further studies are warranted to determine the precise mechanism and role of central versus peripheral GPR3 in the control of temperature, weight and glucose tolerance.

**Figure 6:**
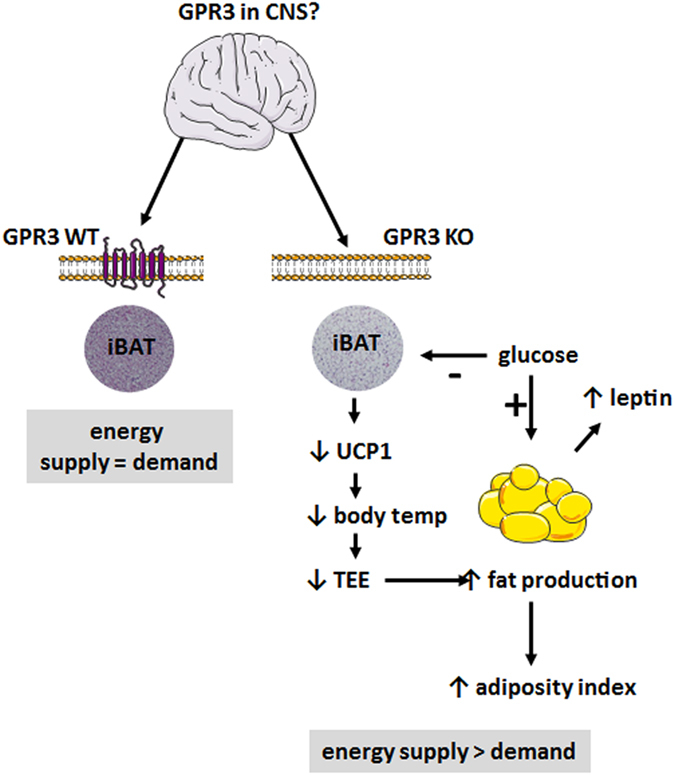
Summary of phenotype changes in GPR3 deficient mice.

In conclusion, as summarized in Fig. 6, the present findings establish a link between age-associated changes in thermogenesis and weight gain *via* the orphan receptor GPR3. Mice lacking GPR3 displayed late-onset obese phenotype due to impaired thermogenic function of iBAT. GPR3 may, therefore, serve as a new pharmacological target in the development of relevant therapeutic intervention to treat obesity in the aging population.

## Methods

### Animals

All animal procedures were approved by the Institutional Animal Care and Use Committee of NIAAA, NIH, and the experiments were carried out in accordance with the accepted guidelines. Data describing phenotype characteristics of GPR3 KO mice are available at Mouse Genome Informatics (MGI strain ID: 101908;

http://www.informatics.jax.org/javawi2/servlet/WIFetch?page=markerDetail&key=17783).

Mice were acquired from the Mutant Mouse Regional Resource Center (MMRRC, stock ID: 011623-UNC). They were on a C57BL/6J genetic background before the arrival and were further bread in our facility according to the MMRRC instructions. They had free access to food (NIH-31 rodent diet) and water and were maintained on a 12:12 light/dark cycle at ambient temperature (21 ± 1 ˚C).

### Genotyping

Genotyping was performed from tail or ear clippings using a REDExtract-N-Amp^TM^ tissue PCR kit (Sigma-Aldrich, USA) according to the MMRRC protocol. The reverse primer (GGAATTAAGCCCTGGTGGACCTAAC) and forward primers (TATCCACTCTCCAAGAACCATCTGG and GGGCCAGCTCATTCCTCCCACTCAT) were used to amplify a 506 bp and 358 bp segment of the wild type and Neo resistance cassette of the mutant mouse, respectively. Products were separated on 0.9% UltraPure^TM^ Agarose gel (Invitrogen, USA) and bands were read on G:BOX scanner (Syngene, USA).

### Experimental protocols

Body weights were recorded every month for 18 months. At the ages of 5 and 12 months, mice were subjected to glucose tolerance and insulin sensitivity tests and were analyzed for substrate utilization by indirect calorimetry. Core body temperature was recorded at ages of 2.5, 5, 12 and 18 months. Distinct subsets of animals were used for hyperinsulinemic euglycemic clamp and cold challenge experiments, as described below.

### Blood and tissue collection

Mice were euthanized by cervical dislocation under isofluorane anesthesia. Trunk blood was collected, clotted within 30 min and transferred on ice. Serum was obtained after centrifugation at 2500 for 15 min at 4 ˚C. Liver, brown and white adipose tissues (subcutaneous, epididymal and perirenal fat pads) were removed, weighed and either snap frozen in liquid nitrogen or fixed in 10% buffered formalin. All tissues were kept at −80 ˚C until analysis. Adiposity index was defined as the ratio of the combined weight of the epididymal, retroperitoneal, and subcutaneous fat pads to total body weight^46^.

### Blood chemistry

Serum triglycerides, total cholesterol, alanine and aspartate aminotransferases (ALT, AST, respectively) were quantified using AMS VegaSys Chemistry Analyzer (Diamond Diagnostics, USA). Serum insulin (Ultra-Sensitive Mouse Insulin ELISA Kit; Crystal Chem Inc), glucagon and leptin (Quantikine ELISA; R&D Systems, USA), adiponectin (Mouse/Rat Adiponectin ELISA Kit, B-Bridge International Inc, USA), peptide C (Mouse C-peptide ELISA Kit; ALPCO Immunoassays, USA), thyroid stimulating hormone (TSH, Lifeome, USA), total thyroxine (T_4_) and triiodothyronine (T_3_) (serum mouse/rat T_3_ and T_4_ total ELISA, Calbiotech Inc. USA) were determined using commercial sandwich ELISA assays in accordance with the manufacturers’ instructions.

### Core body temperature

Core body temperature was recorded in conscious mice at a fixed time at 12 PM using a rectal probe (Ellab Instruments, USA).

### Cold temperature challenge

Thermoregulation was tested as previously described^47^. Briefly, mice were acclimated to the procedure by taking body temperatures once a day for 3 days before the cold temperature challenge. On the day of the experiment, baseline body temperature in mice was recorded at room temperature (22 °C; RT). Animals were then transferred to cold cages (one mouse per cage) without bedding or enrichment and measurements were continued at 4 °C for additional 4 hours. At the end of cold challenge mice were returned to their home cages at RT and subsequent measurements were taken 1 and 4 hours later. Some animals were sacrificed for tissue collection before placing them at 4 ˚C or at the 4^th^ hour of cold challenge. All mice had unlimited access to food and water during the experiment. The cutoff point for the body temperature at which experiment was terminated was set at 34 °C.

### Tissue norepinephrine levels

Tissue norepinephrine (NE) level was quantified by high performance liquid chromatography with electrochemical detection (Millipore Waters 460, USA), as described previously^48^ with modifications. In short, 10–15 mg of brown adipose tissue was homogenized in 500 μL of 0.1 M perchloric acid at 4 ˚C by Precellys-24 with ceramic beads (Bertin Technologies, France). The homogenates were centrifuged, filtered and 20 μL injected onto a 15 cm ion-pair chromatography column (Luna 5 μ C18; Phenomenex; USA). The column was eluted with a mobile phase containing 2.8 g 1-heptanesulfonic acid sodium salt, 0.17 g EDTA, 20 mL triethylamine, dissolved in 2.2 L H2O, pH adjusted to 2.5 with 13 ml 85% phosphoric acid, plus 90 mL acetonitrile. The flow rate was 0.4 mL/min with electrochemical detector sensitivity at 50 nA and applied potential of 0.7 V. Tissue NE peaks were within the linear detectable range of NE standards (0.1–50 ng/20 μL; r^2^ = 0.998).

### Glucose tolerance and insulin sensitivity tests

For glucose tolerance test (GTT), overnight-fasted mice were injected intraperitoneally (ip) with glucose (1.5 g/kg). Blood glucose levels were determined from the tail at 0, 15, 30, 45, 60, 90 and 120 minutes using the glucometer CONTUR TS Blood Glucose Monitoring System (Bayer HealthCare LLC, USA). For insulin sensitivity test (IST), mice were fasted for 6 hours before receiving insulin (0.75 U/kg, ip.; Eli Lilly, DC). Blood glucose levels were determined at the same time points as above.

### Hyperinsulinemic euglycemic clamp

Experiments were performed as described previously^49^, with modifications. Briefly, 5 days before the experiment, the left common carotid artery and the right jugular vein of 5-month-old male GPR3 KO and WT mice were catheterized under isofluorane anesthesia. Following a 5-h period of fasting, clamps were performed on unrestrained, conscious mice. The clamp protocol consisted of a 120-min tracer equilibration period (from t = −120 to 0 min), followed by a 120-min clamp period (from t = 0 to 120 min). A 5-μCi bolus of [3-^3^H]glucose (Perkin Elmer, USA) was given at t = −120 min, followed by a 0.05 μCi/min infusion for 2 h at a pump rate of 0.1 μL/min (CMA Microdialysis, USA). The insulin clamp was begun at t = 0 min with a priming bolus (64 mU/kg) of human insulin (Humulin R; Eli Lilly,USA), followed by an infusion (3.6 mU/kg/min) delivered at a pump rate of 0.1 μl/min from 0 to 120 min. The [3-^3^H]glucose infusion was increased to 0.1 μCi/min for the remainder of the experiment. Specific activity for individual time points did not vary by > 15% from the average specific activity during the last 40 min of the clamp. Euglycemia (∼120–150 mg/dl) was maintained during clamps by measuring blood glucose every 10 min starting at t = 0 min and infusing 40% dextrose as necessary. Blood samples (60 μL) were taken every 10 min from t = 80 to 120 min and processed to determine glucose-specific activity. Mice received saline-washed erythrocytes from donors throughout the experimental period (4 μL/min) to prevent a fall of hematocrit by > 5%. To estimate insulin-stimulated glucose fluxes in tissues, 2-deoxy-d-[1-^14^C]glucose (Perkin Elmer) was bolus administered (10 μCi) at t = 85 min, i.e., 45 min before the end of the experiment. At the end of the clamp, animals were anesthetized with intravenous injection of pentobarbital sodium. Within 5 min, hypothalamus, gastrocnemius muscle from hindlimbs, heart, liver, epididymal, subcutaneous and perirenal fat were removed and frozen until analysis.

To determine [3-^3^H]glucose flux, plasma samples were deproteinized using barium hydroxide and zinc sulfate. The glucose production and disappearance rates were determined using Steele’s non-steady-state equations^50^. Clamp hepatic endogenous glucose production rate was determined by subtracting the glucose infusion rate (GIR) from total glucose turnover (Rd). The glucose uptake by tissues and glycogen synthesis rates were calculated as described previously^51^.

### Tissue histology and staining

Pieces of tissues were fixed in 10% neutral buffered formalin (NBF), embedded in paraffin and sectioned (4 μm) onto glass slides. Routine nuclear and cytoplasmatic staining in both tissues was performed with hematoxylin and eosin, Gills Formula (H&E, Thermo Scientific, USA) and analyzed using an Olympus BX41 microscope (USA). Section of brown adipose tissue were also stained with antibodies against UCP1 (ab10983, Abcam, USA) following antigen retrieval with 0.2% Triton for 15 min (UCP1). The staining was revealed by secondary antibody coupled to FITC (ab6785, Abcam, USA). For fluorescence detection, sections were analyzed using a Zeiss LSM700 confocal microscope. Immunopositivity was quantified using Image J software (NIH Public Domain). Representative images were presented in figures at 10x magnification. Fat cell diameter was measured in 20 different fields from the same paraffin-embedded subcutaneous, epididymal and brown adipose tissue sections.

### Indirect calorimetry, ambulatory activity and food intake

Measurements were conducted in metabolic cages (Oxymax; Columbus Instruments, USA) as described before^46^. The chambers were also equipped with two-dimensional infrared beam sensors (Opto-M3; Columbus Instruments, USA) to measure locomotor activity, and precision weighing balances PL202-S (Mettler Toledo, USA) for continuous monitoring of food intake. Total energy expenditure (TEE) was calculated as VO_2_ × (3.815 + 1.232 × RQ), where RQ is the respiratory quotient (the ratio VCO_2_/VO_2_). Net oxidation rates of fat and carbohydrates were calculated according to the formulae by Simonson and DeFronzo^52^: Fat oxidation = 1.69 × (VO_2_–VCO_2_); Carbohydrate oxidation = 4.57 × VCO_2_–3.23 × VO_2_. Values were normalized with respect to the body weight and adjusted to an effective metabolic body size (kg^0.75^).

### Western blot analyses

Western blotting was performed as described previously with modifications^53^. Tissues were homogenized in RIPA buffer (Thermo Fisher Scientific, USA) containing protein inhibitor cocktail (Roche, USA). Proteins were denatured at 95 ˚C for 5 min, cooled on ice, and 4.5 μg aliquots were fractionated by NuPAGE 10% Bis-Tris gel (NOVEX by Life Technologies, USA) using XT MOPS running buffer (Bio-Rad, USA). The separated fractions were electrotransferred on to a nitrocellulose membrane (Bio-Rad, USA) using NuPAGE transfer buffer (NOVEX by Life Technologies, USA), blocked by incubation for 1 hour at room temperature in TBS-Tween-20 buffer (TBST, Thermo Scientific, USA) containing 5% (w/v) non-fat dry milk (Cell Signaling Technology, USA). Blots were washed 3 times with TBST buffer and incubated overnight at 4 °C with primary antibodies for UCP1 (rabbit polyclonal antibody, diluted 1:500 in 5% BSA in TBST, #ab3298, Abcam, USA), mitochondrial cytochrome c oxidase subunit II (MTCO2; mouse monoclonal antibody diluted 1:500 in 5% BSA in TBST, #ab10983, Abcam, USA) and α-tubulin (rabbit polyclonal antibody diluted 1:1000 in 5% BSA in TBST (#2144, Cell Signaling Technology, USA). The membranes were washed again 3 times in TBST and incubated with with appropriate goat anti-rabbit or anti-mouse IgG diluted 1:500 in 5% milk (#NEF812001EA and #NEF822001EA, respectively; Perkin Elmer, USA) in TBST containing 5% (w/v) non-fat dry milk for 2 hours at room temperature with gentle agitation. Bands were visualized on the Syngene G:BOX scanner using SuperSignal WestPico Chemiluminescence (Thermo Scientific, USA) and quantified with NIH ImageJ software. Protein sizes corresponding to UCP1, MTCO2 and α-tubulin were 33 kDa, 25 kDa and 52 kDa, respectively.

### Real-time PCR

Total mRNA was extracted with Trizol (Invitrogen, USA) and purified by RNeasy Lipid Tissue Mini Kit (Qiagen, USA), followed by DNase I treatment (Invitrogen, USA). Total mRNA was reverse-transcribed using the Iscript cDNA kit (Bio-Rad, USA). Real-time PCR was performed using a StepOne Plus real time PCR system (Applied Biosystems, USA) and the QuantiTect primers (Qiagen, USA) QT00291151, QT00149240, QT00258692, QT00253967, QT01195901, QT00156303, QT01044953, QT01044946, QT00097300, QT00249452, QT00249732, QT00296044 and QT01062656 against mouse stearoyl-CoA desaturase (*Scd1)*, fatty acid synthase (*Fasn*), adrenoceptor beta 1 (*Adrb1*), adrenoceptor beta 2 (*Adrb2*) and adrenoceptor beta 3 (*Adrb3*), peroxisome proliferator-activated receptor gamma, coactivator 1 alpha (*Ppargc1a*), glucose transporter 1 (*Slc2a1*) and 4 (*Slc2a4*), uncoupling protein 1 (*Ucp1*), type 2 iodothyronine deiodinase (*Dio2*), G-protein coupled receptor 3 (*Gpr3*), G-protein coupled receptor 6 (*Gpr6*) and G-protein coupled receptor 12 (*Gpr12*), respectively. Normalization was performed with the QuantiTect primer assay against mouse *Rpl19* (QT01779218) housekeeping gene.

### Statistical analysis

Values are presented as mean ± s.e.m. Statistical data analysis was performed using GraphPad Prism 6 for Windows. Student’s t-test for unpaired data was used for comparison of values between two groups. For multiple groups, ordinary one-way ANOVA analysis of variance, followed by the Dunnett’s post-hoc test was applied. Time-dependent variables were analyzed by two-way ANOVA analysis of variance followed by the Bonferroni’s correction when one group was compared to the control or Tukey’s multiple comparisons test. Differences were considered significant when P < 0.05.

## Additional Information

**How to cite this article**: Godlewski, G. *et al.* Mice lacking GPR3 receptors display late-onset obese phenotype due to impaired thermogenic function in brown adipose tissue. *Sci. Rep.* **5**, 14953; doi: 10.1038/srep14953 (2015).

## Supplementary Material

Supplementary Information

## Figures and Tables

**Table 1:** Serum metabolic markers and whole blood glucose concentration in 12-month-old mice.

	GPR3 WT	GPR3 Het	GPR3 KO
glucose (mg/dL)	143.10 ± 7.83	169.82 ± 16.68	153.67 ± 13.38
insulin (ng/mL)	1.63 ± 0.21	1.78 ± 0.39	1.38 ± 0.16
C peptide (pmol/L)	655.14 ± 48.87	684.65 ± 49.88	632.71 ± 29.88
glucagon (pg/mL)	159.09 ± 32.47	119.81 ± 16.93	163.89 ± 17.45
leptin (ng/mL)	7.81 ± 1.23	12.22 ± 3.25	14.25 ± 2.31*
adiponectin (ng/mL)	21.53 ± 1.16	25.04 ± 1.60	22.60 ± 0.56
triglyceride (mg/dL)	101.67 ± 5.10	134.91 ± 21.92	112.57 ± 8.74
cholesterol (mg/dL)	127.33 ± 4.45	124.00 ± 5.05	136.00 ± 3.49
ALT (U/L)	120.00 ± 6.03	110.18 ± 4.19	122.29 ± 10.06
AST (U/L)	122.67 ± 6.50	109.81 ± 4.45	125.14 ± 9.05

Values are means ± s.e.m. from 10–23 mice. *P < 0.05, compared to GPR3 WT littermates, according to one-way ANOVA followed by Dunnett test.

## References

[b1] Ng M. *et al.* Global, regional, and national prevalence of overweight and obesity in children and adults during 1980–2013: a systematic analysis for the Global Burden of Disease Study 2013. Lancet 384, 766–781 (2014). 24880830 10.1016/S0140-6736(14)60460-8 PMC4624264

[b2] Arterburn D. E., Crane P. K. & Sullivan S. D. The coming epidemic of obesity in elderly Americans. J. Am. Geriatr. Soc. 52, 1907–1912 (2004). 15507070 10.1111/j.1532-5415.2004.52517.x

[b3] Kyrou I. & Tsigos C. Obesity in the Elderly Diabetic Patient Is weight loss beneficial? No. Diabetes Care 32, S403–S409 (2009). 19875589 10.2337/dc09-S348 PMC2811480

[b4] Flier J. S. Obesity wars: molecular progress confronts an expanding epidemic. Cell 116, 337–350 (2004). 14744442 10.1016/s0092-8674(03)01081-x

[b5] Wang Y. C., McPherson K., Marsh T., Gortmaker S. L. & Brown M. Health and economic burden of the projected obesity trends in the USA and the UK. Lancet 378, 815–825 (2011). 21872750 10.1016/S0140-6736(11)60814-3

[b6] Kelly T., Yang W., Chen C. S., Reynolds K. & He J. Global burden of obesity in 2005 and projections to 2030. Int. J. Obes. (Lond) 32, 1431–1437 (2008). 18607383 10.1038/ijo.2008.102

[b7] Vincent G. K. & Velkoff V. A. *The next four decades. The older population in the United States: 2010 to 2050. Current Population Reports.* (2010) Available at: http://www.census.gov/prod/2010pubs/p25-1138.pdf. (Accessed: 26^th^ April 2015).

[b8] Sugatani J. *et al.* Antiobese function of platelet-activating factor: increased adiposity in platelet-activating factor receptor-deficient mice with age. FASEB J. 28, 440–452 (2014). 24100020 10.1096/fj.13-233262

[b9] Wang Z. *et al.* Adult type 3 adenylyl cyclase-deficient mice are obese. PLoS One 4, e6979, 10.1371/journal.pone.0006979 (2009). 19750222 PMC2735775

[b10] Guerra C. *et al.* Brown adipose tissue-specific insulin receptor knockout shows diabetic phenotype without insulin resistance. J. Clin. Invest. 108, 1205–1213 (2001). 11602628 10.1172/JCI13103 PMC209529

[b11] Uehara S. *et al.* Late-onset obesity in mice transgenic for the human renin gene. Int. J. Mol. Med. 11, 723–727 (2003). 12736712

[b12] Bains R. K. *et al.* Visceral obesity without insulin resistance in late-onset obesity rats. Endocrinology 145, 2666–2679 (2004). 15033913 10.1210/en.2003-1608

[b13] Waalen J. & Buxbaum J. N. Is Older Colder or Colder Older? The Association of Age With Body Temperature in 18,630 Individuals. J. Gerontol. A Biol. Sci. Med. Sci. 66, 487–492 (2011). 21324956 10.1093/gerona/glr001 PMC3107024

[b14] Rogers N. H., Landa A., Park S. & Smith R. G. Aging leads to a programmed loss of brown adipocytes in murine subcutaneous white adipose tissue. Aging Cell 11, 1074–1083 (2012). 23020201 10.1111/acel.12010 PMC3839316

[b15] Sellayah D. & Sikder D. Orexin restores aging-related brown adipose tissue dysfunction in male mice. Endocrinology 155, 485–501 (2014). 24248466 10.1210/en.2013-1629

[b16] Eggerickx D. *et al.* Molecular cloning of an orphan G-protein-coupled receptor that constitutively activates adenylate cyclase. Biochem. J. 309, 837–843 (1995). 7639700 10.1042/bj3090837 PMC1135708

[b17] Marchese A. *et al.* Cloning of human genes encoding novel G protein-coupled receptors. Genomics 23, 609–618 (1994). 7851889 10.1006/geno.1994.1549

[b18] Song Z. H., Modi W. & Bonner T. I. Molecular cloning and chromosomal localization of human genes encoding three closely related G protein-coupled receptors. Genomics 28, 347–349 (1995). 8530049 10.1006/geno.1995.1154

[b19] Heiber M. *et al.* Isolation of three novel human genes encoding G protein-coupled receptors. DNA Cell Biol. 14, 25–35 (1995). 7832990 10.1089/dna.1995.14.25

[b20] Harmar A. J. *et al.* IUPHAR-DB: the IUPHAR database of G protein-coupled receptors and ion channels. Nucleic Acids Res. 37, D680–D685 (2009). 18948278 10.1093/nar/gkn728 PMC2686540

[b21] Ye C. *et al.* Identification of a novel small-molecule agonist for human G protein-coupled receptor 3. J. Pharmacol. Exp. Ther. 349, 437–443 (2014). 24633425 10.1124/jpet.114.213082

[b22] Jensen T. *et al.* The identification of GPR3 inverse agonist AF64394; the first small molecule inhibitor of GPR3 receptor function. Bioorg. Med. Chem. Lett. 24, 5195–5198 (2014). 25442311 10.1016/j.bmcl.2014.09.077

[b23] Mehlmann L. M. *et al.* The Gs-linked receptor GPR3 maintains meiotic arrest in mammalian oocytes. Science 306, 1947–1950 (2004). 15591206 10.1126/science.1103974

[b24] Deng J., Lang S., Wylie C. & Hammes S. R. The Xenopus laevis isoform of G protein-coupled receptor 3 (GPR3) is a constitutively active cell surface receptor that participates in maintaining meiotic arrest in X-laevis oocytes. Mol. Endocrinol. 22, 1853–1865 (2008). 18511495 10.1210/me.2008-0124 PMC2505325

[b25] Rios-Cardona D., Ricardo-Gonzalez R. R., Chawla A. & Ferrell J. E. Jr. A role for GPRx, a novel GPR3/6/12-related G-protein coupled receptor, in the maintenance of meiotic arrest in Xenopus laevis oocytes. Dev. Biol. 317, 380–388 (2008). 18381211 10.1016/j.ydbio.2008.02.047 PMC2409273

[b26] Vaccari S., Horner K., Mehlmann L. M. & Conti M. Generation of mouse oocytes defective in cAMP synthesis and degradation: Endogenous cyclic AMP is essential for meiotic arrest. Dev. Biol. 316, 124–134 (2008). 18280465 10.1016/j.ydbio.2008.01.018 PMC2755085

[b27] Norris R. P. *et al.* A G(s)-linked receptor maintains meiotic arrest in mouse oocytes, but luteinizing hormone does not cause meiotic resumption by terminating receptor-G(s) signaling. Dev. Biol. 310, 240–249 (2007). 17850783 10.1016/j.ydbio.2007.07.017 PMC2311505

[b28] Mehlmann L. M. Oocyte-specific expression of Gpr3 is required for the maintenance of meiotic arrest in mouse oocytes. Dev. Biol. 288, 397–404 (2005). 16289135 10.1016/j.ydbio.2005.09.030 PMC1868506

[b29] Freudzon L. *et al.* Regulation of meiotic prophase arrest in mouse oocytes by GPR3, a constitutive activator of the Gs G protein. J. Cell. Biol. 171, 255–265 (2005). 16247026 10.1083/jcb.200506194 PMC2171177

[b30] Lowther K. M., Nikolaev V. O. & Mehlmann L. M. Endocytosis in the mouse oocyte and its contribution to cAMP signaling during meiotic arrest. Reproduction 141, 737–747 (2011). 21411693 10.1530/REP-10-0461

[b31] Ledent C. *et al.* Premature ovarian aging in mice deficient for Gpr3. Proc. Natl. Acad. Sci. USA 102, 8922–8926 (2005). 15956199 10.1073/pnas.0503840102 PMC1150279

[b32] Simpson J. L. Genetic and phenotypic heterogeneity in ovarian failure: overview of selected candidate genes. Ann. N. Y. Acad. Sci. 1135, 146–154 (2008). 18574220 10.1196/annals.1429.019

[b33] Zhou S. *et al.* GPR3 may not be a potential candidate gene for premature ovarian failure. Reprod. Biomed. Online 20, 53–55 (2010). 20158988 10.1016/j.rbmo.2009.10.013

[b34] Thathiah A. *et al.* Beta-arrestin 2 regulates Abeta generation and gamma-secretase activity in Alzheimer’s disease. Nat. Med. 19, 43–49 (2013). 23202293 10.1038/nm.3023

[b35] Thathiah A. *et al.* The orphan G protein-coupled receptor 3 modulates amyloid-beta peptide generation in neurons. Science 323, 946–951 (2009). 19213921 10.1126/science.1160649

[b36] McPartland J. M. & Glass M. Functional mapping of cannabinoid receptor homologs in mammals, other vertebrates, and invertebrates. Gene 312, 297–303 (2003). 12909367 10.1016/s0378-1119(03)00638-3

[b37] Catalano K. J., Bergman R. N. & Ader M. Increased susceptibility to insulin resistance associated with abdominal obesity in aging rats. Obes. Res. 13, 11–20 (2005). 15761159 10.1038/oby.2005.4

[b38] Kim L. J. *et al.* Associations of visceral and liver fat with the metabolic syndrome across the spectrum of obesity: the AGES-Reykjavik study. Obesity (Silver Spring) 19, 1265–1271 (2011). 21183935 10.1038/oby.2010.291 PMC3081537

[b39] Judge M. K. *et al.* Prolonged hyperphagia with high-fat feeding contributes to exacerbated weight gain in rats with adult-onset obesity. Am. J. Physiol. Regul. Integr. Comp. Physiol. 295, R773–780 (2008). 18596107 10.1152/ajpregu.00727.2007 PMC2536857

[b40] Iossa S., Lionetti L., Mollica M. P., Barletta A. & Liverini G. Energy intake and utilization vary during development in rats. J. Nutr. 129, 1593–1596 (1999). 10419996 10.1093/jn/129.8.1593

[b41] Harms M. & Seale P. Brown and beige fat: development, function and therapeutic potential. Nat. Med. 19, 1252–1263 (2013). 24100998 10.1038/nm.3361

[b42] Kusminski C. M. *et al.* MitoNEET-driven alterations in adipocyte mitochondrial activity reveal a crucial adaptive process that preserves insulin sensitivity in obesity. Nat. Med. 18, 1539–1549 (2012). 22961109 10.1038/nm.2899 PMC3745511

[b43] de Jesus L. A. *et al.* The type 2 iodothyronine deiodinase is essential for adaptive thermogenesis in brown adipose tissue. J. Clin. Invest. 108, 1379–1385 (2001). 11696583 10.1172/JCI13803 PMC209445

[b44] Morrison S. F., Madden C. J. & Tupone D. Central control of brown adipose tissue thermogenesis. Front. Endocrinol. (Lausanne) 3, 5, 10.3389/fendo.2012.00005 (2012). PMC3292175 22389645

[b45] Veldhuis J. D. Changes in pituitary function with ageing and implications for patient care. Nat. Rev. Endocrinol. 9, 205–215 (2013). 23438832 10.1038/nrendo.2013.38 PMC3920108

[b46] Tam J. *et al.* Peripheral CB1 cannabinoid receptor blockade improves cardiometabolic risk in mouse models of obesity. J. Clin. Invest. 120, 2953–2966 (2010). 20664173 10.1172/JCI42551 PMC2912197

[b47] Berbari N. F. *et al.* Leptin resistance is a secondary consequence of the obesity in ciliopathy mutant mice. Proc. Natl. Acad. Sci. USA 110, 7796–7801 (2013). 23599282 10.1073/pnas.1210192110 PMC3651481

[b48] Tam J. *et al.* Peripheral cannabinoid-1 receptor inverse agonism reduces obesity by reversing leptin resistance. Cell Metab. 16, 167–179 (2012). 22841573 10.1016/j.cmet.2012.07.002 PMC3832894

[b49] Tam J. *et al.* Role of adiponectin in the metabolic effects of cannabinoid type 1 receptor blockade in mice with diet-induced obesity. Am. J. Physiol. Endocrinol. Metab. 306, E457–E468 (2014). 24381003 10.1152/ajpendo.00489.2013 PMC3923090

[b50] Steele R., Wall J. S., De Bodo R. C. & Altszuler N. Measurement of size and turnover rate of body glucose pool by the isotope dilution method. Am. J. Physiol. 187, 15–24 (1956). 13362583 10.1152/ajplegacy.1956.187.1.15

[b51] Youn J. H., Kim J. K. & Buchanan T. A. Time courses of changes in hepatic and skeletal muscle insulin action and GLUT4 protein in skeletal muscle after STZ injection. Diabetes 43, 564–571 (1994). 8138062 10.2337/diab.43.4.564

[b52] Simonson D. C. & Defronzo R. A. Indirect Calorimetry - Methodological and Interpretative Problems. Am. J. Physiol. 258, E399–E412 (1990). 2180312 10.1152/ajpendo.1990.258.3.E399

[b53] Liu J. *et al.* Functional CB1 cannabinoid receptors in human vascular endothelial cells. Biochem. J. 346, 835–840 (2000). 10698714 PMC1220920

