# Supplementary material for: Mice lacking GPR3 receptors display late-onset obese phenotype due to impaired thermogenic function in brown adipose tissue
Source: Sci Rep. 2015 Oct 12;5:14953. doi: 10.1038/srep14953 (PMC4601089; doi:10.1038/srep14953)
Supplement: Supplementary Information [file srep14953-s1.pdf]

SUPPLEMENTARY INFORMATION

Mice lacking GPR3 receptors display late-onset obese phenotype due to impaired thermogenic function in brown adipose tissue

Grzegorz Godlewski[1*], Tony Jourdan[1], Gergő Szanda[1a], Joseph Tam[1b], Resat Cinar[1], Judith Harvey-White[1], Jie Liu[1], Bani Mukhopadhyay[1], Pal Pacher[2], Fong Ming Mo[1c], Douglas Osei-Hyiaman[1d], George Kunos[1*]

[1]Laboratory of Physiologic Studies and [2]Laboratory of Cardiovascular Physiology and Tissue Injury, National Institute on Alcohol Abuse and Alcoholism of the National Institutes of Health, 5625 Fishers Lane, RM 2S-18, MSC-9413, Bethesda, Maryland 20892-9413, U.S.A.; [a]Current address: Department of Physiology, Semmelweis University, Budapest, Hungary. [b]Current address: Obesity and Metabolism Laboratory, Institute of Drug Research, School of Pharmacy, Faculty of Medicine, The Hebrew University, Jerusalem 91120, Israel. [c]Current address: National Key Laboratory of Nanobiological Technology, Xiangya Hospital, Central South University, 87 Xiangya road, Changsha 4100108, Hunan P.R., China. [d]Current address: Office for Global Education and Career Development, Tokyo Medical and Dental University, 1-5-45 Yushima, Bunkyo-ku, Tokyo 113-8510 Japan.

[*]Corresponding authors: godlewskig@mail.nih.gov and George.Kunos@nih.gov

Laboratory of Physiologic Studies, NIAAA, NIH, Bethesda, MD 20892-9413;

Phone: 301-443-4089; Fax: 301-480-0257

Supplementary Figures S1-6

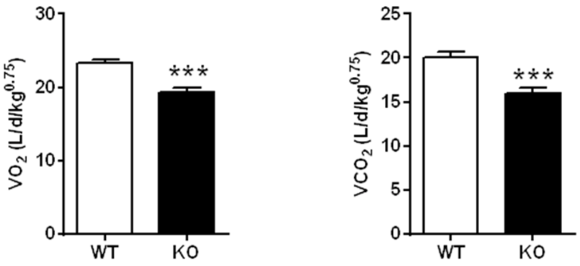

Figure S1. Rates of O_2 consumption (VO2) and CO_2 production (VCO2) in older GPR3 wild type and knockout mice.

The oxygen consumption (VO_2) and carbon dioxide production (CO_2) were monitored by indirect calorimetry older (12-month-old) male wild type (WT) and GPR3 knockout (KO) mice and presented as average of a 24-hour recording period. Values are mean±s.e.m of n=6-8 samples.

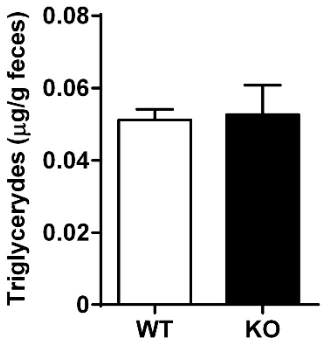

Figure S2. Triglyceride content in feces of adult GPR3 mice.

Values are mean±s.e.m of n=6-9 samples.

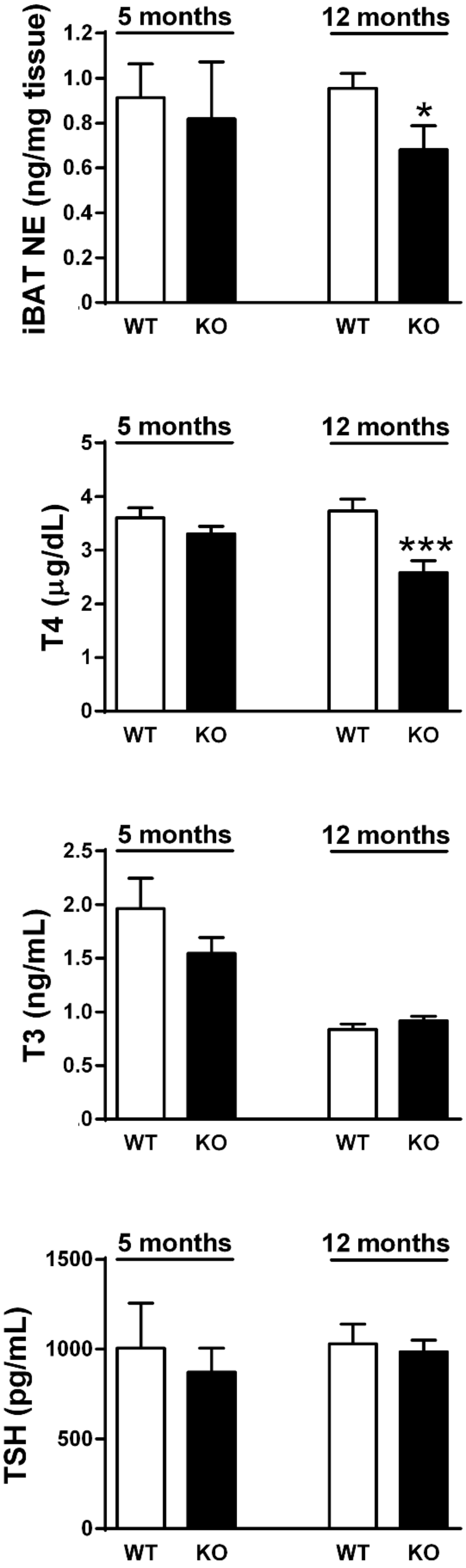

iBAT NE (ng/mg tissue)
5 months
12 months
1.2
1.0
0.8
0.6
0.4
0.2
.0
*
WT
KO
WT
KO
T4 (μg/dL)
5 months
12 months
5
4
3
2
1
0
***
WT
KO
WT
KO
T3 (ng/mL)
5 months
12 months
2.5
2.0
1.5
1.0
0.5
.0
WT
KO
WT
KO
TSH (pg/mL)
5 months
12 months
1500
1000
500
0
WT
KO
WT
KO

Figure S3. Basal iBAT norepinephrine level in brown adipose tissue and serum markers of thyroid function in 5 and 12-month old GPR3 mice at room temperature.

Norepinephrine (NE) level in interscapular brown adipose tissue (iBAT), serum concentration of total thyroxine (T4), total triiodothyronine (T3) and thyroid stimulating hormone (TSH) were measured in ambient conditions in 5 and12-month-old male wild type (WT) and GPR3 knockout (KO) mice as described in Material and Methods. Values are mean±s.e.m of n= 3-8 samples (adult) and n=20-21 samples. *P<0.05, ***P<0.001, compared to age-matched WT littermates.

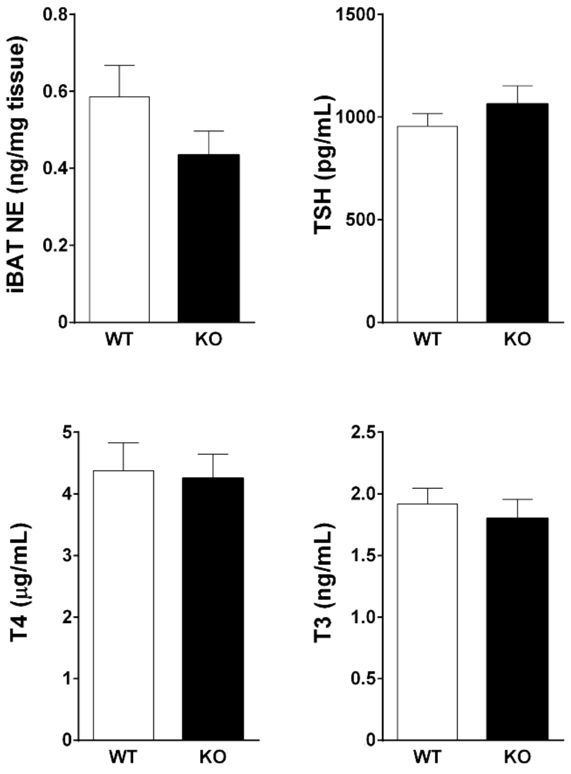

Figure S4. iBAT norepinephrine level and serum markers of thyroid function in 5-month old GPR3 mice during cold challenge.

Interscapular brown adipose tissue (iBAT) and serum samples were obtained from 5-month old male wild type (WT) and GPR3 knockout (KO) mice at the end of the 4-hour cold challenge. Tissue norepinephrine (NE) level and serum concentration of total thyroxine (T4), total triiodothyronine (T3) and thyroid stimulating hormone (TSH) were measured as described in Material and Methods. Values are mean±s.e.m of n= 3-5 samples.

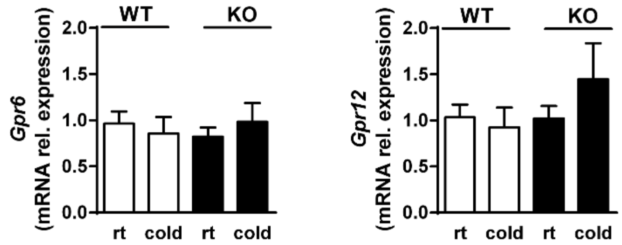

Figure S5. Relative abundance of *Gpr6* and *Gpr12* transcripts in iBAT of 5-month old GPR3 mice at room temperature and during cold challenge

Interscapular brown adipose tissue (iBAT) samples were harvested from 5-month old male wild type (WT) and GPR3 knockout (KO) mice at room temperature (rt) and at the 4th-hour of cold challenge (cold). Genes were corrected via reference *Rpl19* mRNA. Values are mean±s.e.m of n=3-8 samples.

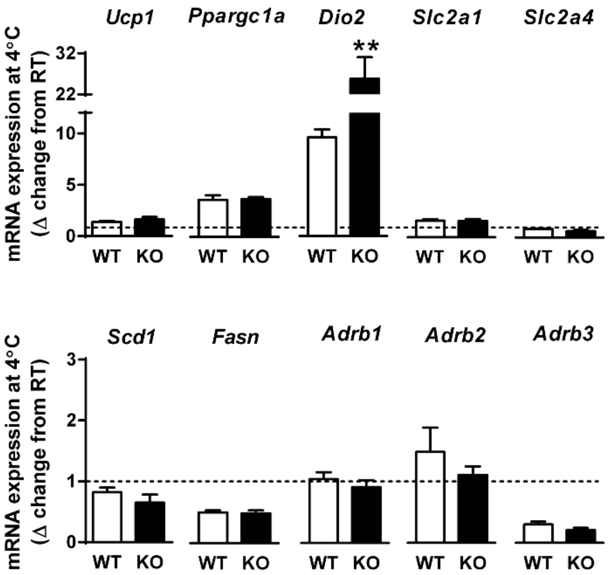

Figure S6. Fold of change in gene expression in iBAT of 5-month old GPR3 mice during cold challenge.

Interscapular brown adipose tissue (iBAT) samples obtained from 5-month old male wild type (WT) and GPR3 knockout (KO) mice at the end of the 4-hour cold challenge. Abundance of target genes was corrected via reference *Rpl19* mRNA. Note, that relative expression of *Ucp1*, *Ppargc1a* and *Dio2* genes differ between GPR3 WT and KO mice at room temperature (see, Figure 5). For the clarity of this figure, the baseline abundance of gene transcript in WT mice at room temperature (RT) was taken as unity (dashed line). Values are mean±s.e.m of n=3-5 samples. $^{**}P<0.01$, compared to the age-matched WT littermates.
